# Influence of Co Content and Chemical Nature of the Co Binder on the Corrosion Resistance of Nanostructured WC-Co Hardmetals in Acidic Solution

**DOI:** 10.3390/ma14143933

**Published:** 2021-07-14

**Authors:** Tamara Aleksandrov Fabijanić, Marin Kurtela, Matija Sakoman, Mateja Šnajdar Musa

**Affiliations:** 1 Faculty of Mechanical Engineering and Naval Architecture, University of Zagreb, Ivana Lučića 5, 10000 Zagreb, Croatia; marin.kurtela@fsb.hr (M.K.); matija.sakoman@fsb.hr (M.S.); 2 Department of Polytechnics, University of Rijeka, Sveučilišna Avenija 4, 51000 Rijeka, Croatia; mateja.snajdar@uniri.hr

**Keywords:** nanostructured hardmetals, Co content, GGIs, chemical nature of Co binder, grain size, electrochemical corrosion resistance, H_2_SO_4_ + CO_2_

## Abstract

The electrochemical corrosion resistance of nanostructured hardmetals with grain sizes *d*_WC_ < 200 nm was researched concerning Co content and the chemical nature of the Co binder. Fully dense nanostructured hardmetals with the addition of grain growth inhibitors GGIs, VC and Cr_3_C_2_, and 5 wt.%Co, 10 wt.%Co, and 15 wt.%Co were developed by a one cycle sinter-HIP process. The samples were detailly characterized in terms of microstructural characteristics and researched in the solution of H_2_SO_4_ + CO_2_ by direct and alternative current techniques, including electrochemical impedance spectroscopy. Performed analysis revealed a homogeneous microstructure of equal and uniform grain size for different Co contents. The importance of GGIs content adjustment was established as a key factor of obtaining a homogeneous microstructure with WC grain size retained at the same values as in starting mixtures of different Co binder content. From the conducted research, Co content has shown to be the dominant influential factor governing electrochemical corrosion resistance of nanostructured hardmetals compared to the chemical composition of the Co binder and WC grain size. Negative values of *E*_corr_ measured for 30 min in 96% H_2_SO_4_ + CO_2_ were obtained for all samples indicating material dissolution and instability in acidic solution. Higher values of *R*_p_ and lower values of *i*_corr_ and *v*_corr_ were obtained for samples with lower Co content. In contrast, the anodic Tafel slope increases with increasing Co content which could be attributed to more pronounced oxidation of the higher Co content samples. Previously researched samples with the same composition but different chemical composition of the binder were introduced in the analysis. The chemical composition of the Co binder showed an influence; samples with lower relative magnetic saturation related to lower C content added to the starting mixtures and more W dissolved in the Co binder during the sintering process showed better corrosion resistance. WC-5Co sample with significantly lower magnetic saturation value showed approximately 30% lower corrosion rate. WC-10Co sample with slightly lower relative magnetic saturation value and showed approximately 10% lower corrosion rate. Higher content of Cr_3_C_2_ dissolved in the binder contributed to a lower corrosion rate. Slight VC increase did not contribute to corrosion resistance. Superior corrosion resistance is attributed to W and C dissolved in the Co binder, lower magnetic saturation, or WC grain size of the sintered sample.

## 1. Introduction

Hardmetals contain tungsten carbide WC particles joined by a binder, most commonly cobalt Co, by a liquid phase sintering process. The properties of the obtained composite derive directly from its constituents; hard and brittle carbides and softer and more ductile binder [1]. By connecting these two components, superior mechanical, physical, and chemical properties are achieved. In recent years, the development of hardmetals is based mainly on the application of ultrafine and nano WC particles (grain size less than 0.5 µm) which require the addition of grain growth inhibitors GGIs to retain the WC grain size in the sintered product. Consequently, a homogeneous microstructure and significantly improved mechanical properties (hardness, wear-resistance, and strength) are achieved. Furthermore, achieving a homogeneous microstructure with a WC grain size in the nano area (<0.2 µm) allows application at higher cutting speeds, lower tolerance, and longer tool life. Due to superior mechanical properties, hardmetals are used in various applications with certain limitations in chemically aggressive environments because of relatively poor corrosion resistance [1].

The corrosion mechanism of conventional WC-Co hardmetals in the neutral and acidic solution is governed by the reduction of the Co binder. At the same time, WC particles are not affected by the corrosion attack [2,3,4]. Zheng et al. found that the binder dissolution started from the center of binder pools in the acid media, independent of binder chemical nature, and spreads to the edges until the binder phase was consumed entirely [4]. Accordingly, it is expected that the corrosion rate will increase with increasing Co content in the starting mixture.

Besides Co binder content, the corrosion mechanisms in hardmetals depend on many other factors such as surface characteristics and integrity, corrosive environment, and hardmetal microstructure [5,6,7]. Hardmetal microstructure, including WC grain size, binder composition/binder chemical nature, grain growth inhibitors GGIs, and porosity, influence the corrosion behavior of hardmetals [3,4,5,6,7,8,9,10]. Researchers have reported different experimental variations concerning the relationship between microstructure and corrosion resistance.

The chemical nature of the Co binder is represented by magnetic saturation. It depends on the C content added to the starting hardmetal mixture and technological processes of consolidation, among which the most important are sintering parameters and atmosphere [11,12,13]. Co binder is advantageous because of the relatively large carbon contents that give the preferred two-phase WC-Co composition, commonly called the carbon window [14,15]. During sintering, the Co binder is alloyed with tungsten (W) and carbon (C); other constituents such as GGIs also add alloying elements to the binder [14]. If a higher amount of tungsten is dissolved in the Co binder, lower relative magnetic saturation values would be obtained [3], and the formation of the brittle η-phase carbides M_6_C and M_12_C would occur in the microstructure of hardmetals. It was found from previous research that hardmetals with lower relative magnetic saturation values show lower values of corrosion current density (*i*_corr_) and critical current density (*i*_crit_) measured by potentiodynamic polarization [2,6,7,16].

Regarding the influence of the WC grain size, different conclusions can be found in the literature. Li Zhang et al. found pseudo-passivation behavior of conventional hardmetals with the WC grain sizes ranging from 1.2 μm to 8.2 μm in sulfuric acid H_2_SO_4_ and better corrosion resistance of the coarse WC grain sizes [3]. On the other hand, Imasato et al. found that the corrosion rate of the WC-Co alloy with a smaller WC grain size was lower than coarse WC grain size both in acid and neutral solution. WC-Co alloy with a smaller WC grain size showed a higher corrosion resistance in the polarization test because of the low current densities of active dissolution and passivated region in the polarization curve [17]. Also, they found that the amount of dissolved metals in neutral and acidic solutions decreased with decreasing WC grain size and carbon content. Most published papers refer to conventional hardmetals, while there is still a lack of results published on nanostructured hardmetals with a WC grain size less than 200 nm.

The paper summarizes long-term research on the corrosion resistance of nanostructured hardmetals. From previous research it was found that the chemical nature of the binder has a more substantial influence on the electrochemical corrosion resistance compared to Co content in the starting mixture in neutral and acidic solution, which was quite surprising and not in line with conventional WC-Co hardmetals [6,7]. The presented research was performed to bring more exact conclusions on the influence of Co content and other microstructural characteristics on the corrosion resistance of nanostructured hardmetals.

## 2. Materials and Methods

Different starting mixtures were prepared to investigate the influence of Co content and chemical nature of the Co binder on the corrosion resistance of nanostructured hardmetals with a grain size *d*_WC_ ˂ 200 nm. WC powder produced by H.C. Starck Tungsten (Goslar, Germany) with an average grain size *d*_BET_ of 95 nm and a specific surface area (BET) of 3.92 m^2^/g, classified as real nanopowder with a grain size less than 100 nm, was used as a carbide phase. Grain growth inhibitors GGIs, vanadium carbide VC and chromium carbide Cr_3_C_2_ were added to the starting mixtures. VC powder has an average grain size *d*_BET_ of 350 nm and a specific surface area (BET) of 3.0 m^2^/g, while Cr_3_C_2_ has an average grain size *d*_BET_ of 450 nm and a specific surface area (BET) of 2.0 m^2^/g. Besides controlling the WC grain growth, VC and Cr_3_C_2_ increase hardness and reduce the rate of corrosion. At the same time, Cr significantly lowers the initial melting point and broadens the melting range, particularly at low carbon levels [12,13,14,16]. The amount of VC and Cr_3_C_2_ differs for each mixture; it was optimized to withhold WC powder size in the sintered samples and increased Co binder content. Half micron cobalt HMP Co, produced by Umicore (Brussels, Belgium), was used as a binder. Three mixtures with different Co content; 5, 10, and 15 wt.%Co were prepared. The consolidation process consisted of powder mixture homogenization in a horizontal ball mill (Zoz GmbH, Wenden, Germany). Compacting was performed by uniaxial die press type CA-NCII 250 (Osterwalder AG, Lyss, Switzerland). Final consolidation to total density was achieved by one cycle sinter-HIP process by furnace FPW280/600-3-2200-100 PS (FCT Anlagenbau GmbH, Sonneberg, Germany) at 1350 °C for 30 min, followed by 100 bars Argon 4.8 pressure for 45 min. The characteristics of the starting mixtures are presented in Table 1.

The goal was to develop fully dense samples with optimal microstructural characteristics with no irregularities such as η-phase in the structure. Previous research found that C and GGIs content and WC grain size have a more substantial influence on the corrosion resistance of nanostructured hardmetals than Co content [6,7]. For the mentioned reason, special care was taken to obtain the optimal and comparable microstructural characteristics of consolidated samples.

The samples were detailly characterized, especially in terms of microstructural characteristics. The characterization of samples consisted of density measurements (Metler Toledo) according to ISO 3369:2006, the specific saturation magnetization (Setaram Instrumentation, Sigmameter) according to D6025, and the coercive field strength measurement (Foerster, Koerzimat 1.096) according to ISO 3326. Diamond disc and pastes were used to grind and polish the samples’ surface before microstructural characterization and electrochemical measurements. Microstructural characterization consisted of porosity, free carbon, and η-phase evaluation. It was performed by comparing the sample’s surfaces with photomicrographs from the standard ISO 4499-4:2016. For that purpose, an optical microscope (Olympus, Shinjuku City, Tokyo, Japan) was used. A field emission scanning electron microscope FESEM (Zeiss, Oberkochen, Germany) was used for WC grain size measurement by the linear intercept method and detection of irregularities such as abnormal growth and WC grains grouping or Co lakes. X-ray diffraction XRD analysis was used to identify the phases present in consolidated samples and exclude the occurrence of η-phase.

After detailed characterization of the samples and determination of optimal microstructural characteristics, the corrosion measurements were performed. The surface of the samples was placed into the corrosion cell filled with H_2_SO_4_ + CO_2_ (pH = 0.6). As reference electrode, saturated calomel electrode SCE (SCHOTT Instruments GmbH, Mainz, Germany) with a potential of + 0.242 V according to the standard hydrogen electrode was selected. Graphite wires were used as a counter electrode. The samples were first researched by direct current techniques DC, the open-circuit potential *E*_corr_, the linear polarization resistance (LPR), and the Taffel extrapolation method. Corrosion potential *E*_corr_ versus SCE was recorded for 30 min. LPR was carried out in the potential range from −0.02 V vs. open circuit potential to 0.02 V vs. open circuit potential with a scan rate of 0.167 mV/s. Tafel extrapolation was conducted in the potential range from −0.25 V vs. open circuit potential to 0.25 V vs. open circuit potential, total points 1001 with a scan rate of 0.167 mV/s. Immediately after the DC techniques, the samples were researched by alternating current (AC) techniques, more precisely by electrochemical impedance spectroscopy (EIS).

The EIS start frequency was 100,000 Hz, and the end frequency was 0.001 Hz; the amplitude was in the range of 10 mV root-mean-square (RMS). The recorded measurements were analyzed by software SoftCorr III (AMETEK Scientific Instruments, Princeton applied research, Berwyn, PA, USA). A convenient electrical equivalent circuit (EEC) was selected by fitting the results of measurements and presented in Nyquist and Bode plots. At each excitation frequency, an imaginary impedance component *Z*im is drawn according to the actual impedance component *Z*re. The impedance and the phase shift curves were plotted against the excitation frequency. Both DC and AC techniques were performed on the potentiostat AMETEK, Princeton applied research, model VersaSTAT3, and the results were recorded and analyzed by software SoftCorr III (AMETEK Scientific Instruments, Princeton applied research, Berwyn, PA, USA).

## 3. Results

### 3.1. Microstructural Characteristics of Consolidated Samples

Characteristics of consolidated samples with different Co content are presented in Table 2. This section may be divided by subheadings. It should provide a concise and precise description of the experimental results, their interpretation, as well as the experimental conclusions that can be drawn.

Full densification was achieved for all samples. The samples are characterized by the lowest possible degree of porosity, A00, B00, and C00, meaning no uncombined/free carbon or η-phase were revealed on the sample’s surface. A high density of samples is related to Co liquid phase, which is spreading onto the surrounding WC particles. Binder propagation is associated with Laplace forces acting along the wetting front between Co binder and WC grains while rearranging the WC particles and reducing the mean distance between neighboring particles, resulting in densification [16,18]. It may be concluded that the WC particles were rearranged, and Co binder filled the micropores between the neighboring WC grains resulting in a theoretical/full density of the samples.

**Figure 1:**
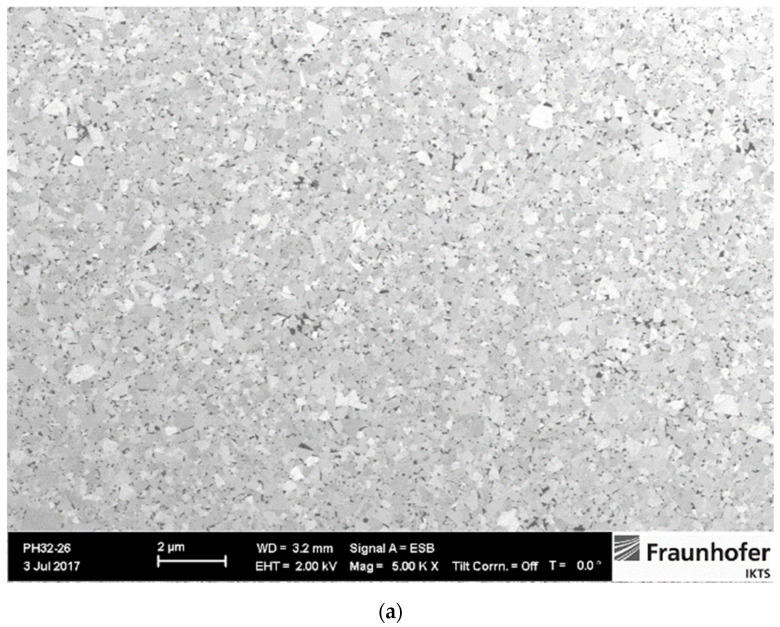

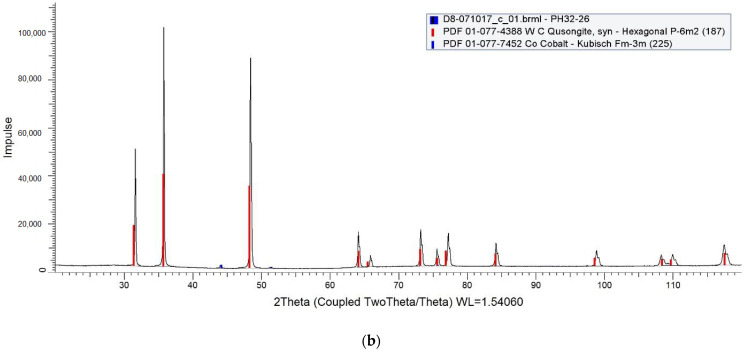
Microstructure and XRD pattern of WC-5Co sample [20]. (**a**) SEM image of microstructure; (**b**) XRD pattern.

**Figure 2:**
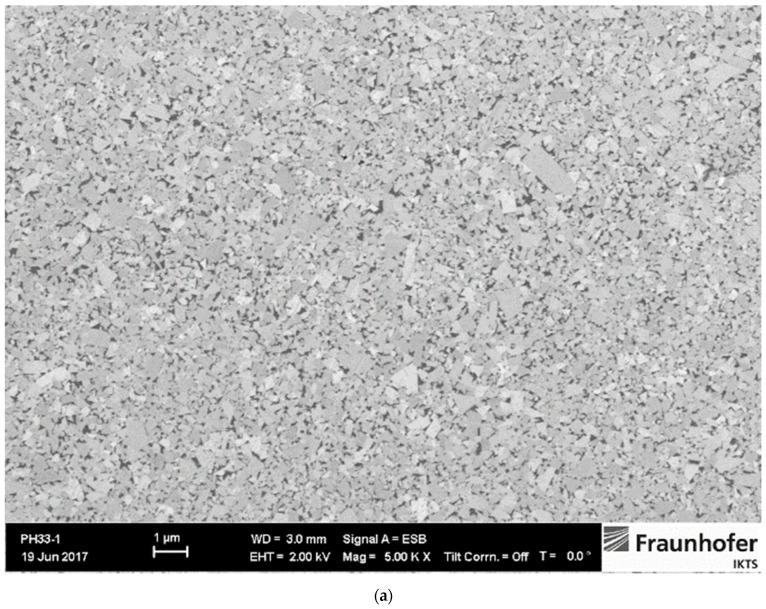

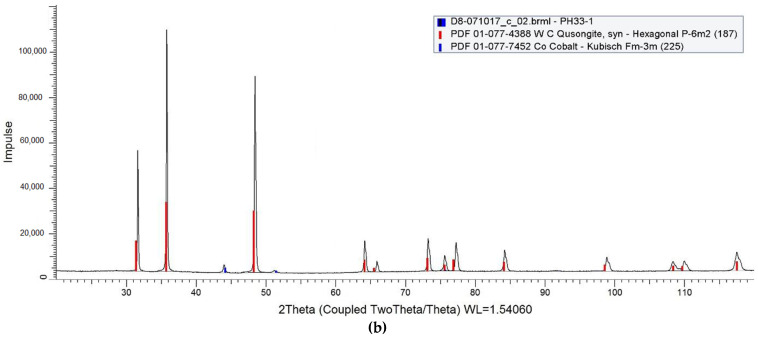
Microstructure and XRD pattern of WC-10Co sample [21]: (**a**) SEM image of microstructure; (**b**) XRD pattern.

**Figure 3:**
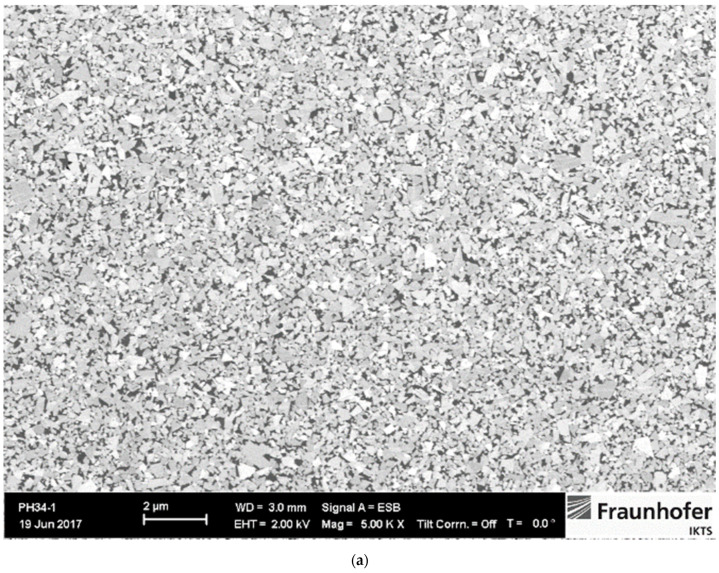

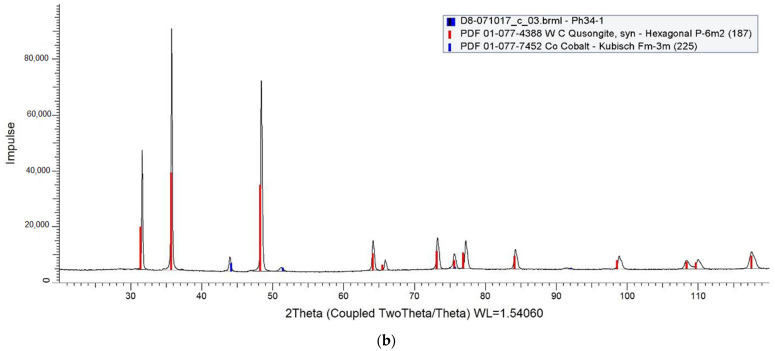
Microstructure and XRD pattern of WC-15Co sample [20]: (**a**) SEM image of microstructure; (**b**) XRD pattern.

The amount of W dissolved in the Co binder phase can be assessed by measuring the magnetic saturation. The saturation value of Co decreases linearly with the addition of tungsten W and is not affected by the carbon content in the solution [19]. Typical relative magnetic saturation/percentage saturation ranges from 80–100% [19], while percentage saturation values higher than 70% indicate two-phase WC-Co microstructure. The highest percentage of 91% was measured for the WC-5Co sample, while 79% was measured for WC-10Co and WC-15Co samples. The two-phase WC-Co microstructure of researched samples is confirmed by optical analysis. Based on coercive force measurement it was estimated the WC grain size. Measured values indicate that all samples fall in the nano range. Two-phase WC-Co microstructure was confirmed by optical analysis, XRD analysis where only WC with the hexagonal crystal structure and Co with FCC cubic crystal structure were identified. Investigation of microstructure revealed homogeneous, uniform distribution of WC grains, without abnormal grain growth due to optimal GGIs content added to the starting mixtures. It was necessary to adjust the content of GGIs for different Co content to achieve a homogeneous and comparable microstructure with retained WC grain size of the starting powders in the sintered samples. Co binder was uniformly distributed, and no Co lakes occurred. Microstructure images and XRD patterns of samples are presented in Figure 1, Figure 2 and Figure 3.

### 3.2. Results of DC Techniques

The results of electrochemical DC techniques are presented in Table 3.

### 3.3. Results of Electrochemical Impedance Spectroscopy EIS

EIS measurements aimed to investigate the corrosion behavior at the interface between the sample surface and electrolyte solution and determine the samples’ corrosion rate. The results are presented in Table 4.

**Figure 4:**
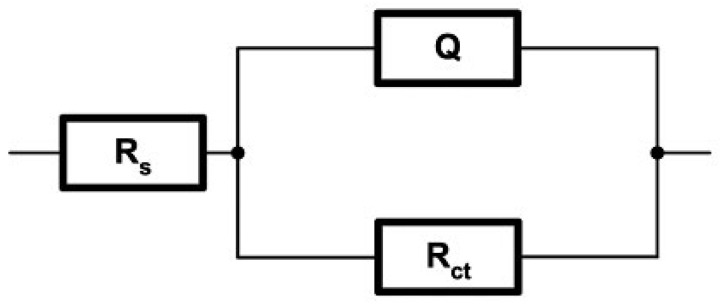
Selected EEC [22].

As already mentioned in Section 2, a convenient and optimal electrical equivalent circuit (EEC) was selected using software SoftCorr III by fitting the measurements’ results and presented in Nyquist and Bode plots. At each excitation frequency, an imaginary impedance component *Z*im is drawn according to the actual impedance component *Z*re. The impedance and the phase shift curves were plotted against the excitation frequency. The selected ECC model is shown in Figure 4.

The same model R(QR), which best corresponds to the processes and reactions on the sample’s surface, was selected for all samples. It is essential to mention that the same EEC model was established in previous research performed on near nanostructured WC-11Co and WC-11Ni samples [22]. The mentioned indicates the repeatability of the corrosion process between nanostructured hardmetal and acidic solutions. The impedance of a constant phase element is defined as:

(1) $$Q={\left[Y{\left(j\omega \right)}^{n}\right]}^{-1}$$

where *Y* and *n* (− 1 ≤ *n* ≤ 1) are constants independent of the angular frequency (*ω*) and temperature. For the value in the range 0.6 < *n* ≤ 1, CPE has the physical meaning of capacitance, an ideal inductor for *n* = −1, and an ideal resistor for *n* = 0.

## 4. Analysis and Discussion

### 4.1. Influence of Co Content on the Corrosion Resistance of Nanostructured WC-Co Hardmetals

**Figure 5:**
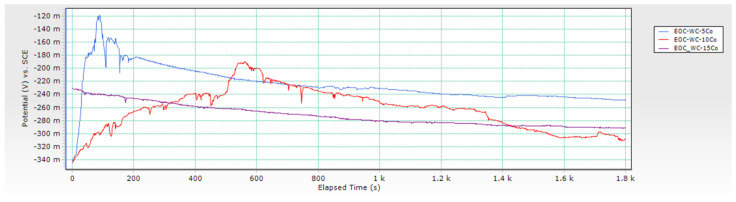
Corrosion potential *E*_corr_ of samples.

**Figure 6:**
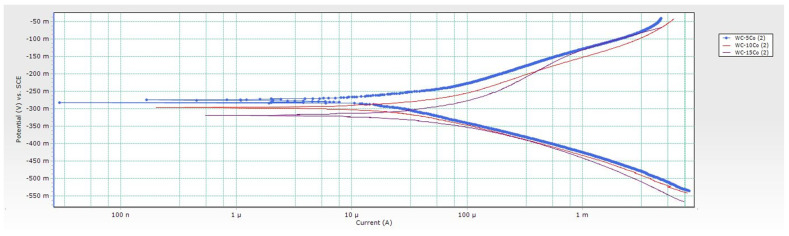
Tafel extrapolation curves.

From conducted research, it can be concluded that the corrosion potential *E*_corr_ of samples changes depending on the Co content. *E*_corr_ of WC-5Co and WC-10Co samples changed from more negative to more positive values indicating that the surfaces of the samples are getting passivated, and a reduction occurred. The corrosion potential curves are unstable and show random fluctuations. A drop from more positive to more negative values was detected for the WC-15Co sample, indicating sample oxidation in contact with the acidic electrolyte and reducing protons at the surface. The changes of *E*_corr_ are not significant, and the corrosion potential variations of each sample occurred in a narrow range. Negative values of *E*_corr_ measured for 30 min in 96% H_2_SO_4_ + CO_2_ were obtained for all samples indicating material dissolution and instability in acidic solution. The *E*_corr_ vs. time curves are presented in Figure 5, and sample Tafel extrapolation curves in Figure 6.

**Figure 7:**
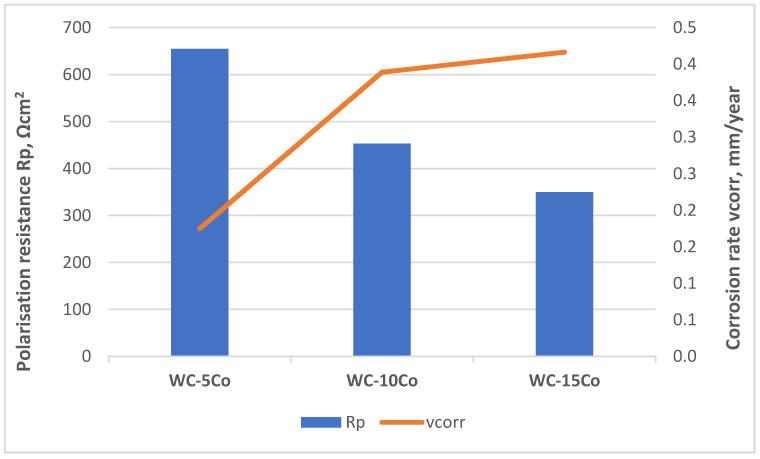
The dependence of *R*_p_ and *v*_corr_ concerning Co content.

Higher values of *R*_p_ and lower values of *i*_corr_ were obtained for samples with lower Co content. Accordingly, the corrosion rate in acidic solution increases with increasing Co content due to selective dissolution of the Co matrix. The cathodic Tafel slopes of samples show a similar trend. In contrast, the anodic Tafel slope increases with increasing Co content which could be attributed to more pronounced oxidation of the higher Co content samples. The dependence of polarization resistance *R*_p_ and corrosion rate *v*_corr_ for different Co contents is presented graphically in Figure 7.

Even though the addition of the refractory metal carbides, VC, Cr_3_C_2_ in the starting mixtures was increased with increasing Co content to maintain the WC powder size, Co content showed stronger influence on the electrochemical corrosion resistance of nanostructured hardmetal samples with optimal microstructural characteristics. The lowest *v*_corr_ of 0.1748 mm/y was obtained for the WC-5Co sample, while the highest *v*_corr_ of 0.4162 mm/y was measured for the WC-15Co sample.

**Figure 8:**
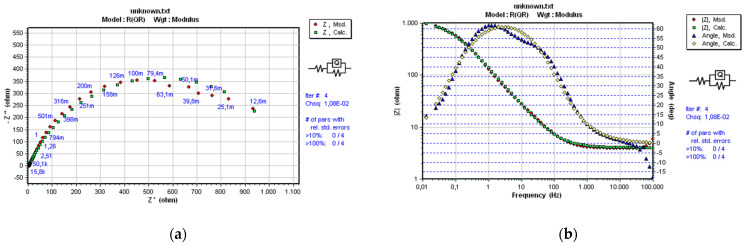
Nyquist (**a**) and Bode (**b**) plots of WC-5Co.

**Figure 9:**
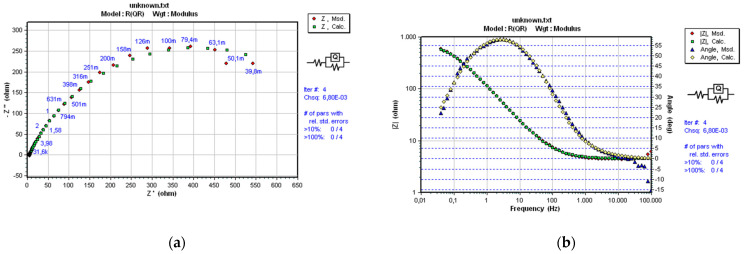
Nyquist (**a**) and Bode (**b**) plots of WC-10Co.

**Figure 10:**
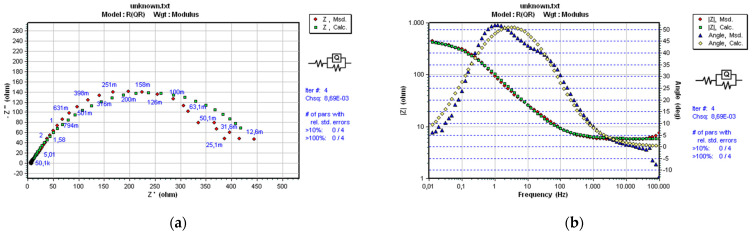
Nyquist (**a**) and Bode (**b**) plots of WC-15Co.

**Figure 11:**
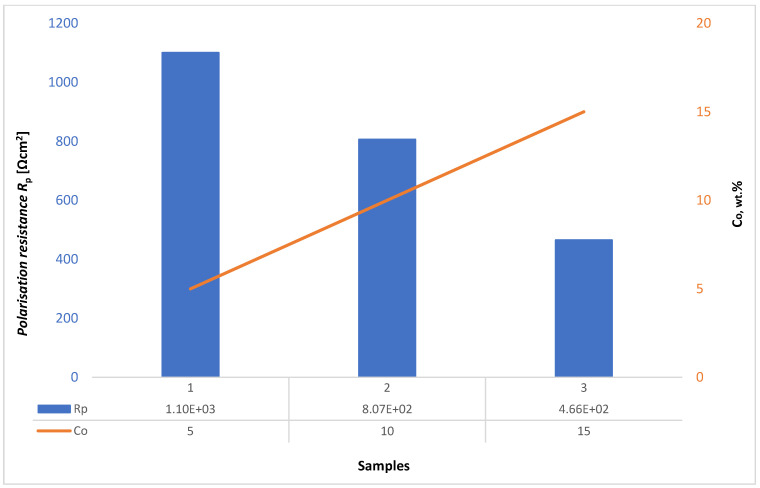
The dependence of *R*_p_ concerning Co content.

Figure 8, Figure 9 and Figure 10 present the Nyquist and Bode diagrams for the WC-5Co, WC-10Co, and WC-15Co samples, obtained using the corresponding EEC simulation model of EIS results. The highest *R*p of 1.101·103 Ωcm^2^ was measured for the WC-5Co sample, followed by *R*p of 8.068·102 Ωcm^2^ measured for the WC-10Co sample, and *R*p of 4.657·102 Ωcm^2^ measured for the WC-15Co sample. Higher *R*p values were detected for the samples with lower Co content, indicating better corrosion resistance (Figure 11) which corresponds to the results obtained by DC linear polarization techniques. It can be seen from Figure 8, Figure 9 and Figure 10 that the radius r of the capacitive semi-circles in the Nyquist plots differ for samples with different Co content. The diameter of the capacitive loop decreased with increasing Co content, indicating better charge transfer resistance on the electrode/electrolyte interface. Subsequently, the decrease in the diameter of the capacitance loop may be ascribed to the weaker protective ability of the sample surface.

Nanostructured hardmetals with optimal microstructural characteristics exhibited behavior similar to previously researched conventional hardmetals with coarser WC grain size. Electrochemical corrosion resistance decreases with increasing Co content in a corrosive, acidic environment due to predominant active Co binder reduction, also known as Co leaching.

### 4.2. Influence of Co Binder Chemical Nature on the Corrosion Resistance of Nanostructured WC-Co Hardmetals

The chemical nature of the Co binder can be characterized by magnetic saturation. It depends on the C content added to the starting hardmetal mixture and consolidation procedure, where sintering parameters and atmosphere have a crucial influence. As referred in the Introduction, it was found from previous research that C content added to the starting mixture can significantly influence the electrochemical corrosion resistance in both neutral (3.5% NaCl with pH = 6.6) and acidic (96% H_2_SO_4_ + CO_2_ with pH = 0.6) environments [6,7]. Comparing WC-5Co samples of the same composition, better corrosion resistance was observed for samples with lower C-added content, lower magnetic saturation, and coarser WC grain size. The opposite behavior governed by the C content and magnetic saturation was noted for WC-15Co samples [7]. Compared to C content, GGIs content, and grain size, the Co content showed less impact on the electrochemical corrosion resistance in both acid and neutral solutions. There was no clear trend of increasing corrosion current densities i_corr_ and decreasing polarization resistance *R*p with increasing Co content typical for conventional hardmetals. Microstructural characteristics, in this case WC grain size, has shown to have a greater influence on the sintered samples corrosion resistance [6,7]. To obtain better insight into the electrochemical corrosion resistance of nanostructured hardmetals, previously researched samples designated as WC-5Co-1 and WC-10Co-1 were introduced in the analysis. WC-5Co-1 and WC-10Co-1 samples were consolidated using same production procedure and characterized by the same methods described in Section 2. The only alteration was the use of lower C and GGIs content added to the starting mixture, which caused different chemical nature of the Co binder and lower values of relative magnetic saturation. Characteristics of the additionally introduced samples and comparison with previously characterized nanostructured hardmetals with *d*_WC_ < 200 nm are presented in Table 5 and Table 6.

**Figure 12:**
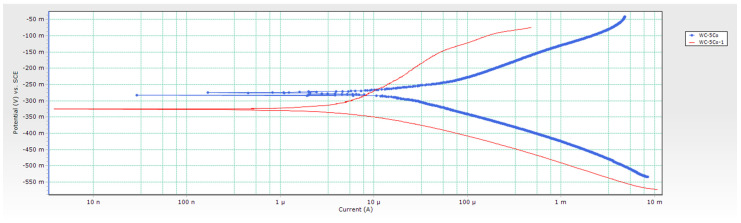
Tafel extrapolation curves of WC-5Co samples with different microstructural characteristics.

As indicated in Table 5, the WC-5Co-1 sample has a significantly lower relative magnetic saturation of 48.0%, attributed to the lower added C content and the different chemical nature of the Co binder. As mentioned, typical relative magnetic saturation/percentage saturation ranges from 80–100%. Saturation percentage values lower than 70% indicate the presence of microstructural irregularity η-phase, confirmed by optical, FESEM, and XRD analysis [6,7]. Previous research found that η-phase most likely enhances the passive layer formation on the sample surfaces, thereby reducing the tendency of sample dissolution and increasing the stability of oxides forming in addition to the existing passive layer on the surface [2,6,7]. The slightly higher measured density of the WC-5Co-1 sample is also associated with η-phase presence in the structure since W_6_Co_6_C or W_3_Co_3_C has higher density when compared to a two-phase WC-Co hardmetal. The coercive force obtained for the WC-5Co-1 sample amounts to 44.9 kA/m and is lower than that of WC-5Co, suggesting a coarser grain size of the WC-5Co-1 sample. Thus, its microstructure can be classified as near nano, in the ultrafine range from 200 to 500 nm. Tafel extrapolation curves of WC-5Co samples are presented in Figure 12.

The WC-5Co-1 sample with lower magnetic saturation value and consequently higher W and C content in the Co binder showed approximately 30% lower corrosion rate, which is in line with previous research. Sutthiruangwong and Mori found that the magnetic saturation related to Co binder composition plays an essential role in the corrosion properties of hardmetals [15,23,24]. F.J.J. Kellner et al. found that electrochemical corrosion resistance of hardmetals is influenced by the W and C diffusion in the Co binder amount which is increased by decreasing the WC grain size [25]. They concluded that W and C dissolved in Co binder during the sintering process stabilize the thermodynamically unstable FCC Co crystal structure at room temperature, the amount of which is increased by an increase of W and C content in the binder. FCC Co is characterized by better corrosion resistance compared to the HCP crystal structure of Co, thermodynamically stable at room temperature [25], when a HCP+FCC Co layer around the HCP Co binder is formed [25]. Their research was performed in an alkaline medium. Still, since in this study the tests were performed in an acidic solution where the lower corrosion resistance of hardmetals is attributed to the dissolution of the HCP Co matrix, these claims could be expected to be more pronounced.

Besides lower magnetic saturation, the WC-5Co-1 sample has a higher content of GGIs, VC, Cr_3_C_2_ in the starting mixture, as presented in Table 5. It is well-known that GGIs are dissolved and distributed among the WC phase and binder during sintering and influence characteristics of hardmetals [26]. Sutthiruangwong and Mori have found that higher corrosion resistance can be assigned to binders that experience higher chromium dissolution rates during sintering [23,24]. Tomlinson and Ayerst found that small additions of Cr_3_C_2_ improve the electrochemical corrosion resistance of hardmetals due to the formation of Cr_2_O_3_ film on the Co binder surface [26]. On the other hand, a small addition of VC in combination with Cr_3_C_2_ decreases the positive Cr_3_C_2_ influence in acidic solution [26]. The WC-5Co-1 sample has a 0.3 wt.% higher content of Cr_3_C_2_, which dissolved in the binder and contributed to a 30% lower corrosion rate than the WC-5Co sample. In this research, it is hard to distinguish which factor has the most substantial influence on electrochemical corrosion resistance. To specify more clearly, WC-10Co samples with different microstructural characteristics and magnetic saturation were compared in Table 6.

**Figure 13:**
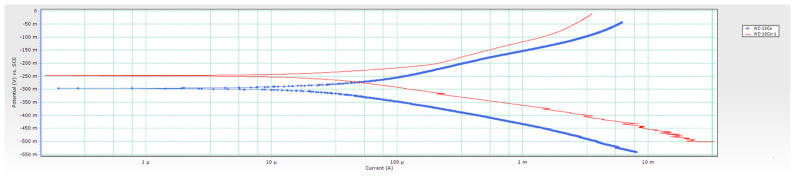
Tafel extrapolation curves of WC-10Co samples with different microstructural characteristics.

The WC-10Co-1 sample has a slightly lower relative magnetic saturation of 74.7% than the WC-10Co sample, which is related to marginally lower C content in the amount of 0.025 wt.% added to the starting mixture. Lower C content resulted in more W dissolved in the Co binder, which, as in the case of the WC-5Co sample, most probably stabilized Co’s thermodynamically unstable FCC crystal structure. Consequently, the WC-10Co-1 sample is characterized by a marginally higher measured density. Density values vary within the two-phase region of the WC-Co phase, and is increased with an increasing amount of W which remains in the Co binder [27]. Sample WC-10Co-1 is located at the lower end of the two-phase WC-Co region in the isothermal part of the WC-Co phase diagram. Therefore, the measured density value is slightly higher than the theoretical density, despite η-phase not detected. The coercive force of WC-10Co-1 amounts to 35.1 kA/m, i.e., lower when compared to WC-10Co, which indicates a coarser grain size of the WC-10Co-1 sample. The same was noted for WC-5Co samples. Tafel extrapolation curves of WC-10Co samples are presented in Figure 13.

The WC-10Co-1 sample with a lower relative magnetic saturation value showed approximately 10% lower corrosion rate. Both microstructures consist of two phases, WC and Co, with no η-phase detected in the microstructure. The difference in GGIs content is relatively small and amounts to an extra 0.13 wt.%VC and 0.03 wt.% Cr_3_C_2_ added to the WC-10Co mixture. It can be concluded that the slight increase of VC wt.% did not contribute to corrosion resistance which corresponds to previously published research. Machio et al. found that small VC addition of 0.4 wt.% increase *i*_corr_ and make hardmetals more sensitive to pitting corrosion due to VC influence on the W dissolution in the Co matrix and formation of (V,W)C layer around the WC grains. VC decreases the dissolution of W atoms in the Co binder during the sintering process and increases the magnetic saturation compared to pure WC-Co hardmetal without GGIs [1,28]. D.S. Konadu et al. found that WC-Co hardmetal possesses nobler corrosion resistance compared to 0.4 wt.%VC containing hardmetal in both HCl and H_2_SO_4_ [1]. Accordingly, better corrosion resistance in this research may be related to W and C dissolution in the Co binder, magnetic saturation, or WC grain size in the sintered sample.

## 5. Conclusions

The following conclusions can be drawn from the conducted research:

(1) Fully dense nanostructured hardmetals with a WC grain size *d*_WC_ ≤ 200 nm were developed utilizing the single-cycle sinter-HIP process. For different Co contents, a homogeneous microstructure of equal and uniform grain size without microstructural defects in the form of carbide agglomerates, abnormal grain growth, or Co lakes was successfully obtained.
(2) The importance of GGIs content adjustment was established as a key factor of obtaining a homogeneous microstructure with WC grain size retained at the same values as in starting mixtures of different Co binder content.
(3) The Co content in the starting mixture proved to have a significant influence on the electrochemical corrosion resistance of nanostructured hardmetals in acidic solution. A noticeable trend of polarization resistance *R*_p_ decrease, and current density *i*_corr_ and corrosion rate *v*_corr_ increase has been established with increasing Co content. Nanostructured hardmetals with the grain size *d*_WC_ ˂ 200 nm showed the same corrosion behavior as coarser grain-size conventional WC hardmetals depending on the Co content.
(4) The chemical composition of the Co binder showed a significant influence. Samples with lower relative magnetic saturation related to lower added C content and more W dissolved in the Co binder showed better corrosion resistance. Significant differences in magnetic saturation for samples with the same Co content lead to more pronounced differences in the corrosion rates. A slight difference in magnetic saturation and WC grain size changed the Taffel curves.
(5) Co content was shown to be the dominant influential factor governing electrochemical corrosion resistance of nanostructured hardmetals when compared to the chemical composition of the Co binder and WC grain size. Samples with lower Co content exhibited lower corrosion rates.
(6) The slight increase of GGIs content, Cr_3_C_2_, and VC did not improved the corrosion resistance significantly for the samples with the same Co content. Higher content of Cr_3_C_2_ dissolved in the binder contributed to a lower corrosion rate. Slight VC increase did not contribute to corrosion resistance. Superior corrosion resistance is attributed to W and C dissolved in the Co binder, lower magnetic saturation, or WC grain size of the sintered sample.

## Figures and Tables

**Table 1:** The characteristics of the starting mixtures.

Mixture	Starting WC Powder	Grain Size *d*_BET_, nm	Specific Surface, m^2^/g	Co, wt.%	GGI, wt.%
WC-5Co	WC DN 4-0 (H.C. Starck)	95	3.92	5	0.3% VC 160 (H. C. Starck) 0.5% Cr_3_C_2_ 160 (H. C. Starck)
WC-10Co				10	0.5% VC 160 (H. C. Starck) 0.75% Cr_3_C_2_ 160 (H. C. Starck)
WC-15Co				15	0.75% VC 160 (H. C. Starck) 1.13% Cr_3_C_2_ 160 (H. C. Starck)

**Table 2:** Characteristics of consolidated samples.

Sample	Density, g/cm^3^	Relative Density, %	Magnetic Saturation, µTm^3^/kg	Rel. Magnetic Saturation, %	Coercive Force, kA/m	ISO Porosity			*d*_WC_, nm
						A	B	C	
WC-5Co	14.91	100.0	8.4	92	52.0	A00	B00	C00	187
WC-10Co	14.31	100.0	14.8	79	40.0	A00	B00	C00	198
WC-15Co	13.84	100.0	22.3	79	37.0	A00	B00	C00	192

**Table 3:** Electrochemical DC techniques results.

Sample	*T*s [°C]	*E*_corr_ vs. SCE [mV]	*R*_p_ [Ωcm^2^]	*β*_a_ [mV/dec]	*β*_c_ [mV/dec]	*i*_corr_ [μA/cm^2^]	*v*_corr_ [mm/y]
WC-5Co	20 ± 2	−249	654.5	75.31	90.37	20.7	0.1748
WC-10Co	20 ± 2	−308	452.8	98.34	97.67	36.6	0.3888
WC-15Co	20 ± 2	−291	349.9	120.19	86.95	50.8	0.4162

*T*s—measured temperature; *E*_corr_—corrosion potential; *R*_p_—polarization resistance; *β*a—a slope of anodic Tafel curve; *β*c—a slope of cathodic Tafel curve; *i*_corr_—corrosion current density; *v*_corr_—corrosion rate.

**Table 4:** Electrochemical impedance spectroscopy EIS technique results.

Sample	*T*s [°C]	*R*_s_ [Ωcm^2^]	*Q*	*n*_1_	*R*_p/_*R*_ct_ [Ωcm^2^]
WC-5Co	20 ± 2	4.022	1.761·10^−3^	0.745	1.101·10^−3^
WC-10Co	20 ± 2	4.504	2.213·10^−3^	0.725	8.068·10^−2^
WC-15Co	20 ± 2	5.797	2.552·10^−3^	0.683	4.657·10^−2^

*T*s—measured temperature; *R*_s_—solution resistance between the working electrode and the reference electrode in a three-electrode cell; *Q*—Constant Phase Element (CPE); *n*_1_—constant; *R*_ct_—polarization resistance or resistance to charge transfer on the electrode/electrolyte interface.

**Table 5:** Comparison of WC-5Co samples with different characteristics.

Sample	GGI, wt.%	Added C, wt.%	Density, g/cm^3^	*ρ*, %	Relative Magnetic Saturation, %	Coercive Force, kA/m	*v*_corr_ [mm/y]
WC-5Co-1	0.41%VC, 0.80% Cr_3_C_2_	0.150	14.96	100	48.0	44.9	0.1181
WC-5Co	0.30%VC, 0.50% Cr_3_C_2_	0.275	14.91	100	92.0	52.0	0.1748

**Table 6:** Comparison of WC-10Co samples with different microstructural characteristics and magnetic saturation.

Sample	GGI, wt.%	Added C, wt.%	Density, g/cm^3^	*ρ*, %	Relative Magnetic Saturation, %	Coercive Force, kA/m	*v*_corr_ [mm/y]
WC-10Co-1	0.37%VC, 0.72% Cr_3_C_2_	0.225	14.35	100	74.7	35.1	0.3463
WC-10Co	0.5%VC, 0.75% Cr_3_C_2_	0.250	14.32	100	79.0	40.0	0.3888
